# Impact of diatomite addition on lead immobilization in air pollution control residues from a municipal solid waste incinerator

**DOI:** 10.1007/s11356-021-17349-x

**Published:** 2021-11-09

**Authors:** Hiroki Kitamura, Masato Ueshima, Seungki Back, Noppharit Sutthasil, Hirofumi Sakanakura, Tomonori Ishigaki, Masato Yamada

**Affiliations:** 1 Material Cycles Division, National Institute for Environmental Studies, 16-2 Onogawa, Tsukuba, Ibaraki 305-8506 Japan; 2 R&D Center, Yoshino Gypsum Co., LTD, 2-1-1 Kohoku, Adachi-ku, Tokyo, 123-0872 Japan

**Keywords:** Municipal solid waste, Air pollution control residues, Metal immobilization, Diatomite, Pozzolanic reaction

## Abstract

Air pollution control (APC) residues, which are known to be the byproducts of incineration treatment, exhibit a high leaching potential of toxic metals. Calcium silicate hydrate (C-S–H), which is a major hydration product of hardened cement and immobilizes toxic metal, can be formed by the reaction of Ca with pozzolanic Si in a highly alkaline environment. Toxic metals might be immobilized by the addition of pozzolanic material to APC residues (instead of using cement), which is a Ca source and provides an alkaline condition. In this study, diatomite, which mainly comprises amorphous silica (SiO_2_·nH_2_O), was investigated as a pozzolanic material for Pb immobilization in APC residues obtained from a municipal solid waste incinerator. APC residues were cured with and without the addition of diatomite at different temperatures. When diatomite was added to APC residues, pozzolanic phases such as C-S–H gel were formed via the consumption of Ca(OH)_2_ and CaClOH. Compared to APC residues cured without diatomite, the leaching of Pb decreased by 99% for APC residues cured for 14 days with 10% diatomite at 70 °C. The results of sequential chemical extraction showed that water-soluble Pb in APC residues was reduced from 10.3% to nearly zero by the pozzolanic reaction. Consequently, the leaching amount of Pb dropped below 0.3 mg/L (Japanese criteria for landfill disposal). Overall, these experiments provide promising results regarding the possibility of using diatomite for pretreating APC residues.

## Introduction

Municipal solid waste incineration (MSWI) has been initiated for hygienic control (Gohlke and Martin 2007). New landfill construction is complicated by the “not in my backyard” (NIMBY) syndrome and limited availability of space (Asakura et al. 2010). Owing to volume and mass reduction, incineration is the most common treatment for municipal solid waste (MSW) (Lombardi et al. 2015). Even after reduction, MSWI residues (bottom ash and air pollution control (APC) residues) continue to be byproducts of incineration treatment. Bottom ash and APC residues account for 10–15%, and 2–3% of the weight of MSW, respectively (Chang and Wey 2006). Furthermore, MSWI residues contain toxic metals (Jung et al. 2004). In particular, the leachability of toxic metals in APC residues is higher than that in bottom ash (Kida et al. 1996; Song et al. 2004). Because of the high leaching potential of toxic metals, pretreatments of APC residues are required in many countries to prevent the release of toxic metals into the environment (Quina et al. 2008). The available treatments for APC residues can be categorized into three groups: (1) physical or chemical separation, (2) solidification/stabilization (S/S), and (3) thermal treatment (Van Der Sloot et al. 2001). Separation treatments aim to reduce the toxic metals and/or soluble salts from the residues via washing and leaching among other processes (Quina et al. 2008; Zacco et al. 2014). Solidification/stabilization (S/S) treatments produce a material with physical (specific surface area, porosity, etc.), mechanical (durability, mechanical strength, etc.), and chemical properties that can immobilize the hazardous species in waste (Zacco et al. 2014). Thermal treatments reduce leaching and the volume of residues, thereby producing a material that is suitable for reuse (Zacco et al. 2014).

Among these treatments, cement-based S/S is commonly used for metal immobilization in APC residues worldwide (Polettini et al. 2001; Benassi et al. 2016). Calcium silicate hydrate (C-S–H), which features high microporosity and a large surface area, is a major hydration product of hardened cement pastes (Gougar et al. 1996). In cement-based S/S processes, C-S–H gel is formed by pozzolanic reactions between amorphous silica (SiO_2_·nH_2_O) and calcium hydroxide (Ca(OH)_2_) (Dermatas et al. 2005). C-S–H gel can immobilize toxic metals via sorption, incorporation, and encapsulation (Chen et al. 2009). Based on the mechanism of the pozzolanic reaction, cement is not a necessity for C-S–H gel formation. If C-S–H gel can be formed using inexpensive materials instead of cement, the treatment cost would be reduced. Dissolved amorphous silica in the presence of Ca ions is required for the formation of C-S–H gel. The solubility of amorphous silica increases at alkaline pH (Alexander et al. 1954). APC residues usually contain high amounts of Ca as a sorbent and reactant for the removal of acidic components in exhaust gas (Wang et al. 2010). APC residues often show high pH due to the presence of alkaline Ca compounds (Shim et al. 2005; Karlfeldt Fedje et al. 2010). Owing to the high Ca content and alkaline pH, the addition of amorphous silica to APC residues may induce C-S–H gel formation via pozzolanic reactions for metal immobilization. Recently, diatomite has received increasing attention as a natural pozzolanic material owing to its high amorphous silica content (Kastis et al. 2006; Ergün 2011), relative abundance (Xu et al. 2016), and low cost compared to Portland cement (Li et al. 2019). Diatomite is a sedimentary rock composed mainly of skeletons of individual diatoms, which are unicellular microscopic plants. In cement-based S/S processes, other cement hydrates such as ettringite (3CaO·Al_2_O_3_·3CaSO_4_·32H_2_O) are also formed. Ettringite can incorporate toxic metals into its structure (Ca^2+^, Al^3+^, and SO_4_^2−^ sites) by substitution reactions (Gougar et al. 1996). Because the main components of APC residues are similar to those in cement (Liu et al. 2018), cement hydrates can be formed in APC residues without cement addition (Montagnaro et al. 2003). Although it is unclear whether other cement hydrates except C-S–H gel contribute to metal immobilization in actual systems (Geysen et al. 2004), the mineralogical characteristics of APC residues might have the potential for metal immobilization without using cement.

In this context, this study aims to investigate the feasibility of using diatomite on metal immobilization (particularly for Pb) in APC residues instead of using cement. Lead immobilization, which occurs due to C-S–H gel formation after diatomite addition, was mainly investigated along with the impacts of secondary mineral formation. In this study, the conditions for Pb immobilization, such as doses of diatomite, curing temperature, and curing time, were investigated. The effect on Pb immobilization was evaluated by the Japanese leaching test to determine the leachabilities of toxic metals from wastes and to determine whether the waste is hazardous. The mineralogical characteristics of APC residues and the chemical form of Pb which changed following diatomite addition were also investigated.

## Materials and methods

### Air pollution control residue sample and diatomite

An APC residue sample was collected from the bag filter of a stoker-type incinerator in Japan. The incineration capacity was 80 tons/day. The incinerator is equipped with a dry scrubbing system using slaked lime (Ca(OH)_2_) to neutralize the acidic components (HCl and SOx) in the flue gas. The unreacted slaked lime and/or the reaction products in the APC residue sample could be used as Ca sources for the pozzolanic reaction.

Amorphous silica was essential for the pozzolanic reaction in this study. Diatomite containing amorphous silica in the presence of Ca in APC residues promotes C-S–H gel formation. Although reagent-grade diatomite is sometimes calcinated owing to the removal of impurities such as organic matter and carbonate compounds from raw diatomite (Ren et al. 2014), the amorphous silica phase is transformed into a crystalline phase such as cristobalite (SiO_2_) by calcination (Zheng et al. 2018). For this reason, non-calcinated diatomite (DiatomaceousEarth.com, USA) was selected as a pozzolanic material for the formation of C-S–H gel.

### Characterization of air pollution control residue sample and diatomite

The chemical compositions of the APC residue sample and diatomite were analyzed using a wavelength dispersive X-ray fluorescence spectrometer (XRF: ZSX Primus II, Rigaku Co., Japan). The XRF results were obtained in oxide form and then converted into elemental form (mass%). The mineral compositions were determined via X-ray diffraction (XRD; MultiFlex, Rigaku Co., Japan). The measurement conditions for XRD analysis were as follows: 40 kV accelerating voltage, 26 mA current, 5–75° 2 θ scanning range, 0.01° step, and 1°/min scan speed. Morphological characteristics were observed using a field emission scanning electron microscope (FE-SEM, JSM-7800F, JEOL Ltd., Japan) under low-vacuum conditions. The elemental composition of each APC residue particle was analyzed by energy-dispersive X-ray (EDX) attached to SEM (SEM–EDX: X-Max^N^ 50, Oxford Instruments, UK). The leachability of regulated toxic metals (As, Cd, Cr, Pb, and Se) from APC residues was investigated using the Japanese leaching test. First, 20 mL of distilled water was added to 2 g of APC residue in a polypropylene bottle (liquid to solid ratio = 10). The bottle was shaken horizontally at a rate of 200 times per minute with an amplitude of 5 cm for 6 h. After shaking, the bottle was centrifuged at 3000 rpm for 20 min. The supernatant was filtered through a cellulose acetate membrane filter with a 0.45-µm pore size. The concentrations of toxic metals in the leachate were measured by inductively coupled plasma mass spectrometry (ICP-MS; 720 ICP-MS, Agilent Technologies, USA) and inductively coupled plasma optical emission spectroscopy (ICP-OES: 7500CX ICP-MS, Agilent Technologies, USA).

### Immobilization of lead in air pollution control residues by curing

Diatomite accounting for 0%, 5%, and 10% with respect to weight was added to 30 g of APC residues at different doses. Subsequently, 22.5 mL of distilled water was added. The mixture was kneaded using a mixer (DLC-1 J, Cuisinart, USA) to create a paste. The paste was wrapped in a thin plastic sheet and stored in a sealed plastic bottle to maintain moist conditions, and subsequently cured at 25, 50, or 70 °C for 14 days. After 1, 2, 4, 7, and 14 days, the cured APC residues were dried in a desiccator using a drying agent (silica gel) at 25 °C. After drying, the cured APC residue was subjected to XRD analysis, SEM observation, and the leaching test described in the “Characterization of air pollution control residue sample and diatomite” section. The chemical form of Pb was estimated by sequential chemical extraction modified from the method suggested by Tessier et al. (1979). Raw APC residues and cured APC residues, which yielded the lowest leaching concentration of Pb in this study, were subjected to sequential chemical extraction. Table 1 shows the fractional patterns with the extraction methods. The sequence of fractional patterns is as follows: (F1) water-soluble, (F2) exchangeable, (F3) bound to carbonates, (F4) bound to metal oxides, (F5) bound to organic matter, and (F6) residual. The concentration of Pb in each fractionated extract solution was measured, and the results were given as the ratio of the total amount of Pb in each solution. Sequential chemical extraction suggested by Tessier et al. (1979) was originally developed for soils and sediments. Although sequential chemical extraction has been widely applied to MSWI residues, its results may not necessarily reflect associations with the target fractions (Wan et al. 2006; Funatsuki et al. 2012).

**Table 1:** Procedures of sequential chemical extraction

Fraction		Procedure
F1	Water soluble	10 g (dry weight) of sample was shaken for 6 h at room temperature with 100 mL of purified water
F2	Exchangeable	The residue from F1 was shaken for 18 h at room temperature with 100 mL of 1.0 mol/L CH_3_COONH_4_
F3	Bound to carbonates	The residue from F2 was shaken for 18 h at room temperature with 100 mL of 1.0 mol/L CH_3_COONa adjusted to pH 5 with CH_3_COOH
F4	Bound to metal oxides	The residue from F3 was shaken for 18 h at 85 °C with 100 mL of 0.2 mol/L NH_2_OH·HCl with CH_3_COOH in 25 vol/vol%
F5	Bound to organic matters	The residue from F4 was shaken for 2 h at 85 °C with 30% H_2_O_2_ adjusted to pH 2 with 0.02 mol/L HNO_3_. After cooling, 100 mL of 1.78 mol/L CH_3_COONH_4_ with HNO_3_ in 11.1 vol/vol% was added and the sample was agitated for 30 min
F6	Residual	2.5 mL of concentrated HNO_3_ and 7.5 mL of concentrated HCl were added to 0.2 g (dry wight) of residue from F5 and heated at 120 °C covered with a watch glass. The sample was filtrated using a 5B filter in order to obtain supernatant

## Results and discussion

### Basic characteristics of diatomite and air pollution control residue sample

**Fig. 1:**
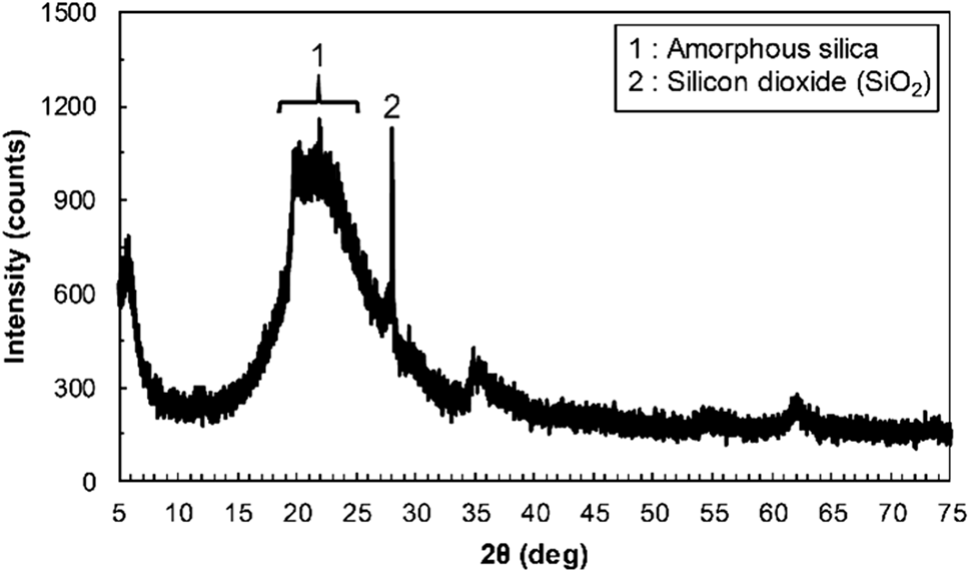
X-ray diffraction (XRD) pattern of diatomite

**Fig. 2:**
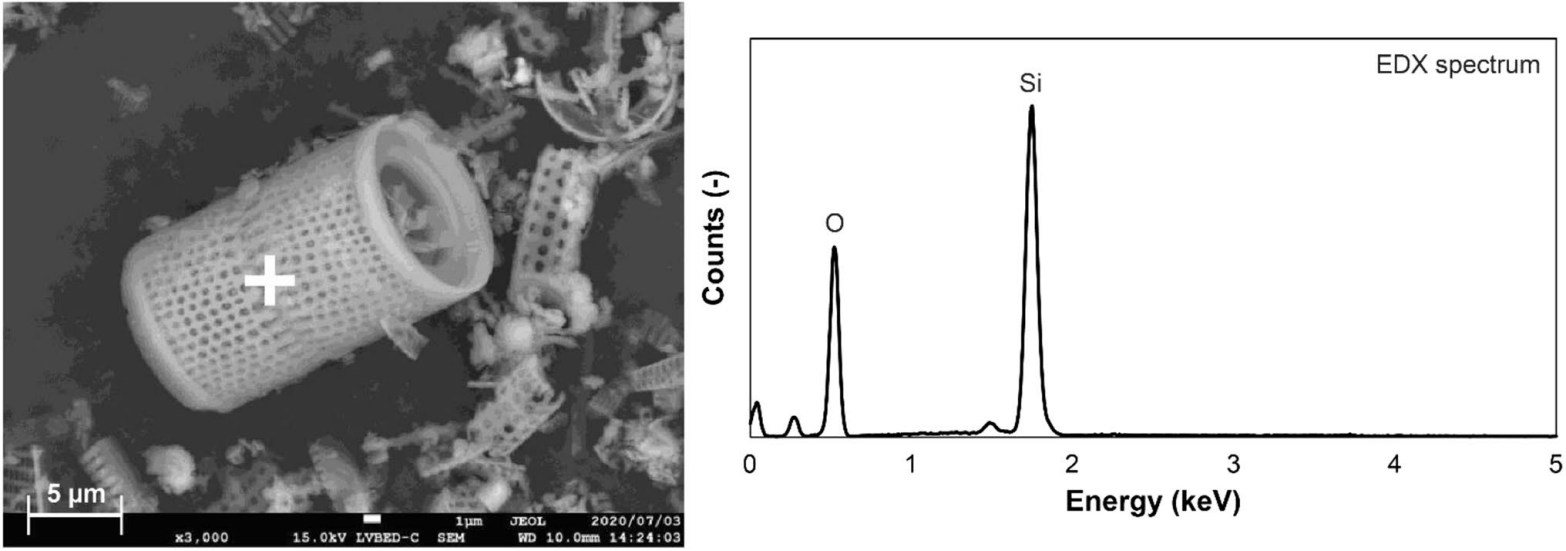
Scanning electron microscopy (SEM) image and elemental spectrum of diatomite (white cross mark; analysis point)

Table 2 shows the chemical compositions of diatomite determined by XRF analysis. The results showed that diatomite consists mainly of Si (42.5 mass%) and minor compositions such as Al and Fe (2.77 mass% and 0.96 mass%, respectively). Figure 1 shows the XRD pattern of diatomite. A broad peak (hump) of amorphous silica and a peak of silicon dioxide were observed as reported by other studies (Sierra et al. 2010; Ergün 2011). Figure 2 shows the backscattered electron image of diatomite and the elemental spectrum. Diatomite shows a cylindrical and porous structure containing Si.

**Table 2:** Chemical compositions of diatomite determined by X-ray fluorescence (XRF) analysis

Elements	Si	Al	Fe	Ca	Mg	Ti	Na	K	S	P	Cl	O
mass %	**42.5**	**2.77**	**0.96**	**0.62**	**0.37**	**0.21**	**0.15**	**0.16**	**0.013**	**0.0090**	**0.0092**	**52.1**

**Fig. 3:**
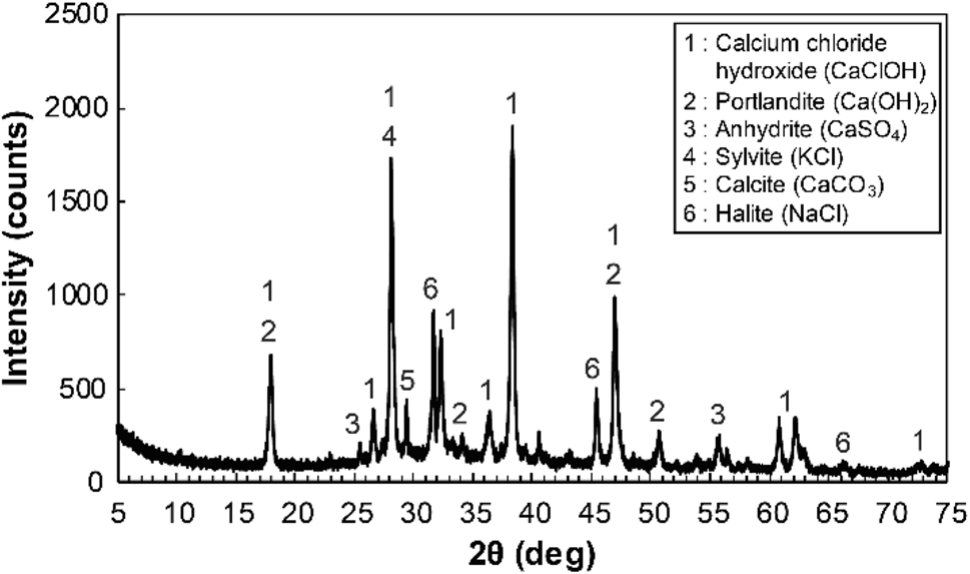
XRD pattern of the air pollution controlled (APC) residue sample

According to XRF analysis, Ca and Cl were the two dominant elements of the APC residue sample, accounting for 36.4% and 32.7% of the mass, respectively (Table 3). Toxic metals such as Cd, Cr, and Pb were also identified as minor elements. Figure 3 shows the XRD pattern of the APC residue sample. The results showed that the APC residue sample mainly comprised Ca-bearing minerals and inorganic salts such as sylvite (KCl) and halite (NaCl). The high Ca content determined by XRF analysis was attributed to Ca-bearing minerals such as portlandite (Ca(OH)_2_), calcium chloride hydroxide (CaClOH), anhydrite (CaSO_4_), and calcite (CaCO_3_), whereas the high Cl content was attributed to CaClOH, KCl, and NaCl. Portlandite is an unreacted slaked lime that is sprayed for acidic gas treatment. Calcium chloride hydroxide is formed during the acidic gas (HCl) neutralization process (Eqs. (1)–(3)) (Jozewicz and Gullett 1995). Anhydrite is also formed by the neutralization of SOx (Eq. (4)) (Bodénan and Deniard 2003). Calcite is formed from portlandite via carbonation by atmospheric moisture and CO_2_ (Eq. (5)) (Weibel et al. 2016). Substances with high vapor pressures and low boiling points, such as alkali and volatile metals, undergo volatilization and transfer to flue gas during the incineration process (Weibel et al. 2016). Cooling of flue gas promotes the formation of alkali metal chlorides such as KCl and NaCl because the chemical potential of Cl, K, and Na exceeds that of volatile metals (Chiang et al. 1997; Durlak et al. 1997).

**Table 3:** Chemical compositions of the air pollution control (APC) residue sample determined by XRF analysis

Elements	Ca	Cl	Na	S	K	Si	Al	Mg	Fe	Zn	Ti
mass %	36.4	32.7	3.93	1.34	1.86	0.62	0.40	0.38	0.33	0.31	0.23
Elements	P	Br	Cd	Pb	Ba	Cu	Ag	Sr	Mn	Cr	O
mass %	0.14	0.17	0.12	0.10	0.051	0.049	0.053	0.030	0.023	0.016	20.2

1 $${\mathrm{Ca}(\mathrm{OH})}_{2}{+ 2\mathrm{HCl}\to \mathrm{CaCl}}_{2}{+ 2\mathrm{H}}_{2}\mathrm{O}$$

2 $${\mathrm{Ca}(\mathrm{OH})}_{2}{+\mathrm{ HCl}\to \mathrm{CaClOH }+\mathrm{ H}}_{2}\mathrm{O}$$

3 $${\mathrm{Ca}(\mathrm{OH})}_{2}{+\mathrm{ CaCl}}_{2}\to 2\mathrm{CaClOH}$$

4 $${\mathrm{Ca}(\mathrm{OH})}_{2}{+\mathrm{ SO}}_{2}{+ 1/2\mathrm{ O}}_{2}\to {\mathrm{CaSO}}_{4}{+\mathrm{ H}}_{2}\mathrm{O}$$

5 $${\mathrm{Ca}(\mathrm{OH})}_{2}{+\mathrm{ CO}}_{2}\to {\mathrm{CaCO}}_{3}{+\mathrm{ H}}_{2}\mathrm{O}$$

**Fig. 4:**
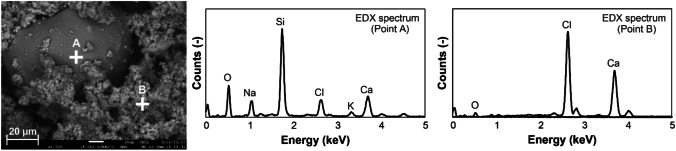
SEM image and elemental spectra of APC residue particles (white cross mark; analysis point)

Figure 4 shows the backscattered electron image of the APC residue particles and elemental spectra. According to SEM observations, the APC residue sample showed larger particles (several tens of micrometers) with smooth surfaces and aggregates of smaller particles (several micrometers) as the main morphological structures. According to SEM–EDX analysis, larger particles contained Ca, O, and Si among other elements. These glasses are produced from melt droplets during the MSWI process (Bayuseno and Schmahl 2011). Aggregates of smaller particles, which consist mainly of Ca, Cl, and O, are considered unreacted slaked lime (Ca(OH)_2_) and the reaction product (CaClOH) from the acidic gas neutralization process.

Table 4 shows the results of the leaching tests of APC residues. The pH value of the leachate was 12.0, which is close to the pH value of the saturated Ca(OH)_2_ solution (12.4). The results also exhibited the leaching of a considerable amount of Pb (11.7 mg/L). Among the regulated toxic metals in Japan, volatile metals such as Cd and Pb are enriched in APC residues (Fernandez et al. 1992). The distribution of As in MSWI residues varies according to the combustion conditions (Jung et al. 2004). The concentration of Se is very low compared to that of other volatile metals (Jung et al. 2004). Chromium mainly remains in the bottom ash because Cr compounds are not thermally mobile during incineration (Jung et al. 2004). Among these regulated toxic metals, Pb is the most problematic owing to its high leachability (Quina et al. 2010). This study focused on Pb immobilization from the viewpoint of leaching potential.

**Table 4:** The pH and leaching concentrations of toxic metals from raw APC residues (mean ± standard deviation, *n* = 3)

Regulated toxic metals	pH ( −)	As (mg/L)	Cd (mg/L)	Cr (mg/L)	Pb (mg/L)	Se (mg/L)
Leaching concentration	12.0 ± 0	0.004 ± 0.0001	N.D	0.24 ± 0.003	11.7 ± 0.09	N.D
Regulated limit		0.3	0.3	1.5	0.3	0.3

*N.D.* below detection limit

### Impact of curing of air pollution control residues on lead immobilization

**Fig. 5:**
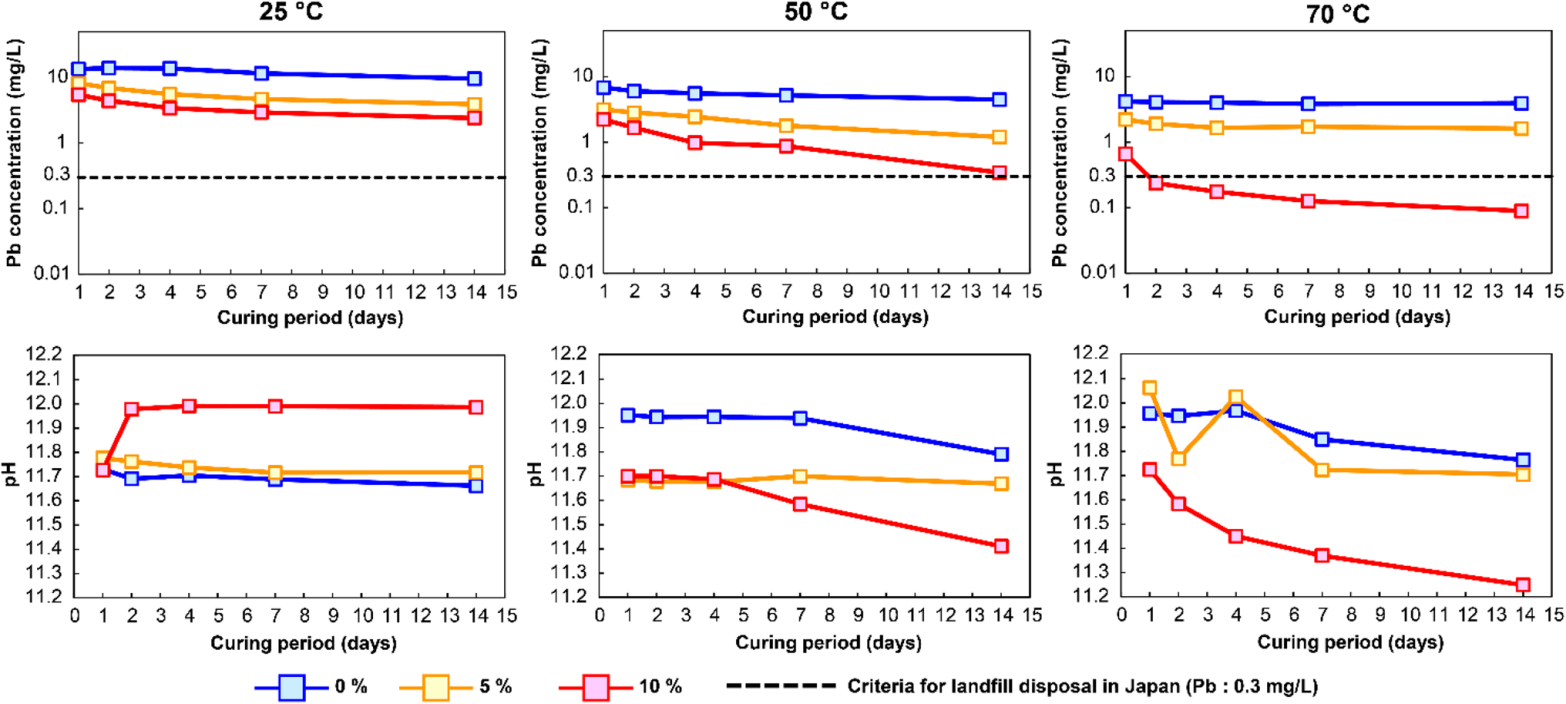
Leaching concentration of lead (Pb) from cured APC residues (diatomite addition: 0, 5, and 10%)

Figure 5 shows the leaching concentration of Pb from cured APC residues and the pH of the leachate. Since an increase in curing temperature accelerates the early hydration reaction of cementitious materials (Greenberg 1961; Escalante-García and Sharp 1998), Pb leaching from cured APC residues decreased as curing time and temperature increased. At each curing temperature, Pb leaching from the cured APC residues decreased as diatomite doses increased. In APC residues cured without diatomite (blue lines in Fig. 5), the leaching amount of Pb after 14 days of curing was reduced by 18–67% from raw APC residues (11.7 mg/L). This means that Pb leaching was decreased by factors other than diatomite addition. The possible reasons are discussed in the “Changes in mineralogical characteristics” section with XRD results. A comparison of Pb leaching by APC residues cured with and without diatomite can quantitatively clarify the impact of diatomite addition on Pb immobilization. The leaching amount of Pb was further reduced by 67–90% and 80–99% by curing with the addition of 5% and 10% diatomite, respectively (orange and red lines in Fig. 5). Consequently, the leaching amount of Pb dropped below 0.3 mg/L (Japanese criteria for landfill disposal) after 14 days of curing with the addition of 10% diatomite at 70 °C.

**Fig. 6:**
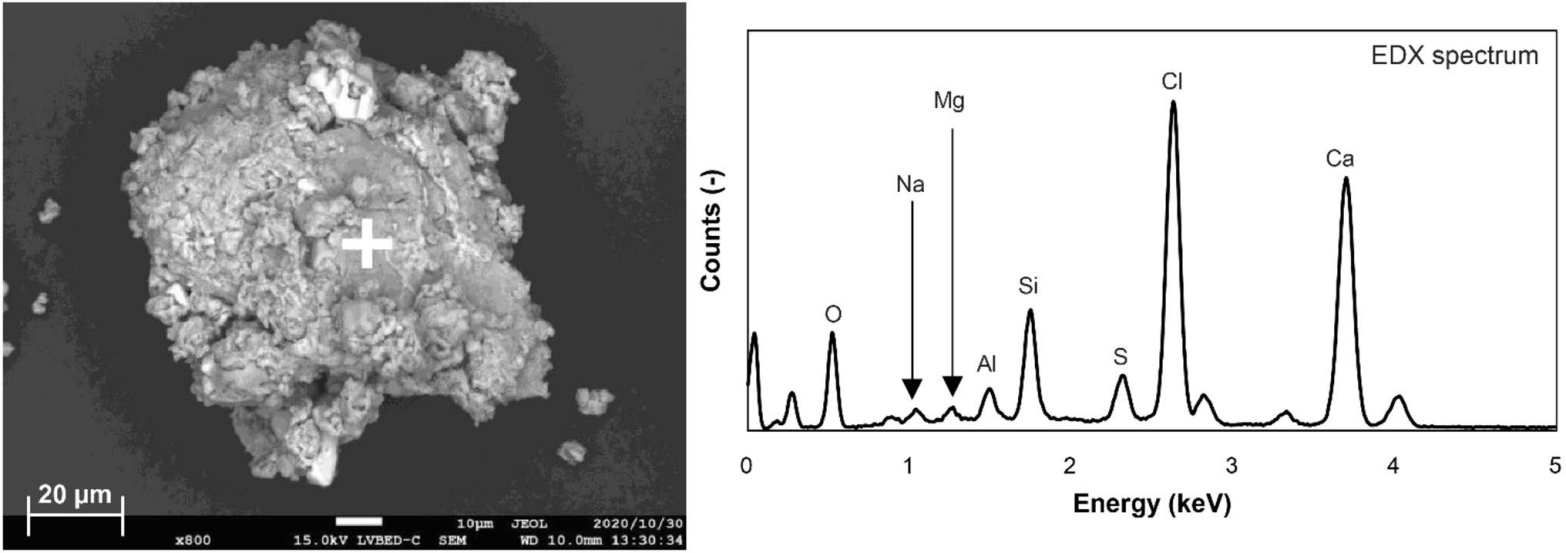
SEM image and elemental spectrum of cured APC residues following the addition of 10% diatomite at 70 °C for 14 days (white cross mark; analysis point)

In these curing experiments, the pH of the leachate decreased slightly in certain cases. This is mainly ascribed to the reaction of Ca(OH)_2_ with dissolved diatomite at alkaline pH and its consumption by the pozzolanic reaction (Dermatas et al. 2005). Figure 6 shows the backscattered electron image and the elemental spectrum of 14-day cured APC residue particles at 70 °C. According to SEM observations, the cylindrical and porous structure of diatomite disappeared, indicating that diatomite reacted with Ca(OH)_2_ (Sierra et al. 2010). Additionally, APC residue particles appeared to be well connected and formed an aggregate containing Ca, Si, and other elements via the pozzolanic reaction.

**Fig. 7:**
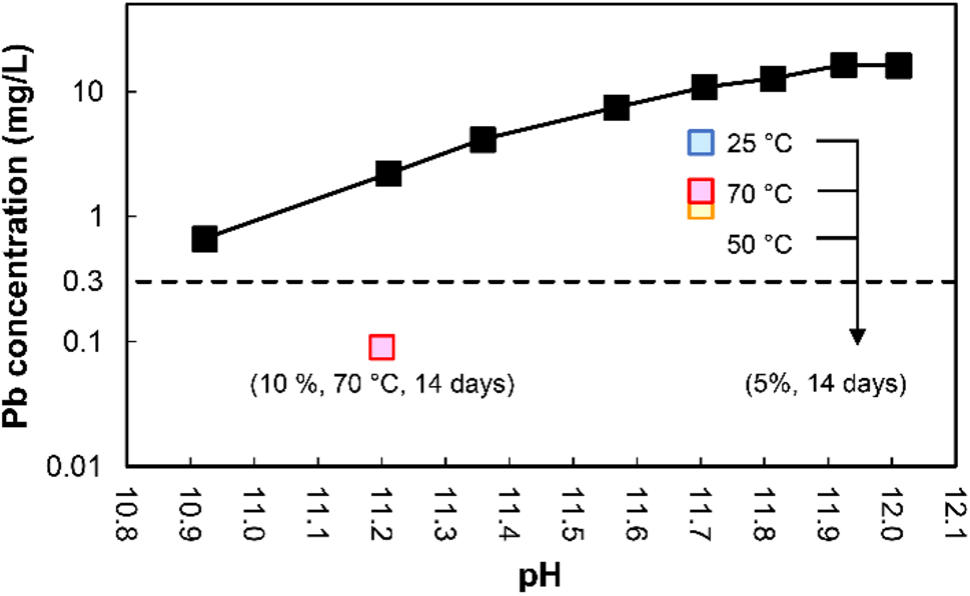
Leaching concentration of Pb from raw APC residues at adjusted pH

Lead solubility heavily depends on pH owing to amphoteric characteristics (Kosson et al. 1996). Thus, the impact of the pozzolanic reaction on Pb immobilization remains unclear owing to the decrease in pH due to curing. To confirm the impact of pH change on Pb solubility, the concentration of Pb leached from raw APC residues at adjusted pH was measured using leaching tests. In this leaching test, an extractant with different amounts of nitric acid (HNO_3_) and distilled water was used to adjust the pH at the endpoint. Figure 7 shows the result of the leaching test at pH adjusted to approximately 11–12. First, the leached concentration of Pb at a pH of 11.7 was confirmed because all pH values of the leachate of 14-day cured APC residues following the addition of 5% diatomite were approximately 11.7, as shown in Fig. 5. The results showed that the leached concentration of Pb from raw APC residues at a pH of 11.7 was 10.8 mg/L, whereas all leached concentrations of Pb from 14-day cured APC residues following the addition of 5% diatomite were significantly lower. The leached concentration of Pb at a pH 11.2 was also confirmed for comparison with 14-day cured APC residues following the addition of 10% diatomite at 70 °C (0.09 mg/L). The results showed a significant decrease in Pb leaching at a pH of 11.2 (2.2 mg/L). However, the leaching concentration of Pb still exceeded 0.3 mg/L (Japanese criteria for landfill disposal).

Instead of using cement, these results show the feasibility of using diatomite for Pb immobilization in APC residues. Although 10% diatomite was added to APC residues in this study, the volume is supposed to be lower than that of cement-based S/S (Sun et al. 2016). The treatment cost is also supposed to be lower than that with cement-based S/S as the average cost of diatomite is one-tenth of the average price of Portland cement (Li et al. 2019). Notably, the global anthropogenic CO_2_ emissions generated by the production of Portland cement have reached 9% (Li et al. 2020). In contrast, the CO_2_ emissions of mining and processing of diatomite are significantly lower than those of manufacturing Portland cement (Li et al. 2020). Chemical agents are also used for the metal immobilization of APC residues to avoid volume increases. Organic chelating agents are preferred in Japan (Mizutani et al. 2000; Sakanakura 2007) and previous studies showed that they immobilize toxic metals better than other chemical agents (Jianguo et al. 2004; Zhang et al. 2016). In contrast to this advantage, COD components derived from organic chelating agents induce long-term leachate treatment at landfill sites (Higuchi 2015). Using diatomite would reduce the use of chemical agents while suppressing volume increases compared to those with cement. Thus, the treatment proposed in this study seems to be low-cost and environmental-friendly.

To apply this method to other APC residue samples, the relationships among Pb leaching, diatomite characteristics, and curing conditions should be further investigated. For example, the reactivity of diatomite with Ca(OH)_2_ (pozzolanic reactivity) plays an important factor in this treatment because the Ca/Si ratio affects the physical and chemical properties of C-S–H gel. The cation exchange capacity and specific surface area of C-S–H gel increase as the Ca/Si ratio decreases (Suda et al. 2015; Bernard et al. 2021). Luxan et al. (1989) proposed a method to evaluate the pozzolanic activity by measuring the change in the electric conductivity of a saturated Ca(OH)_2_ solution with pozzolanic materials. In another saturated lime test, pozzolanic activity is quantitatively determined by directly measuring Ca(OH)_2_ consumed by pozzolanic materials (Mohammed 2017). These indicators of pozzolanic activity seem to be useful in appropriately determining the dose of diatomite and the curing conditions for Pb immobilization. Therefore, the method for controlling the Ca/Si ratio should be investigated further.

### Changes in mineralogical characteristics

**Fig. 8:**
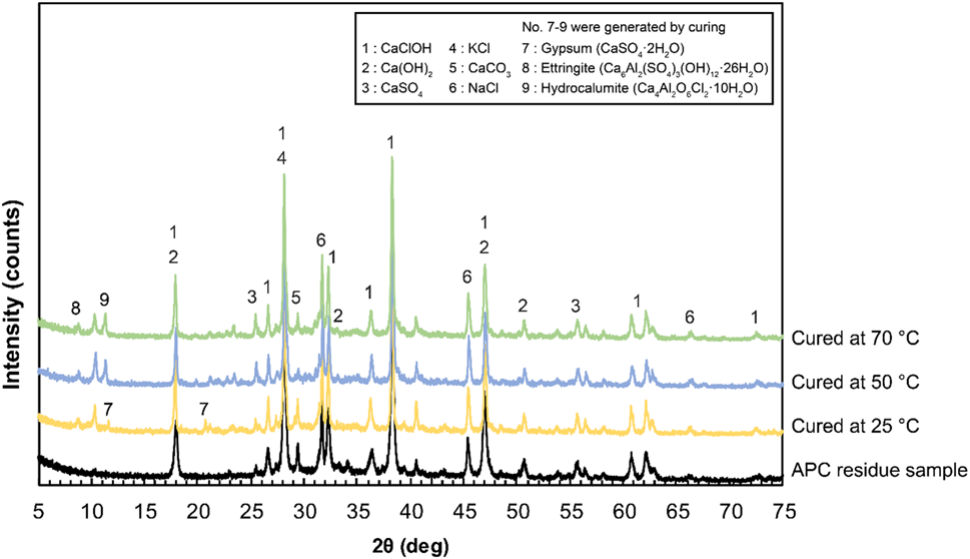
XRD patterns of raw and cured APC residues without diatomite addition

Figure 8 shows the XRD patterns of the APC residues cured without diatomite as compared to the raw APC residues. Several new peaks appeared at approximately 10° 2θ after curing. Gypsum (CaSO_4_**·**2H_2_O) was formed in APC residues cured at 25 °C. Ettringite (Ca_6_Al_2_(SO_4_)_3_(OH)_12_·26H_2_O) was formed in all the cured APC residues. These secondary minerals can be formed in APC residues via hydration reaction (Kitamura et al. 2018). Hydrocalumite (known as Friedel’s salt (Ca_4_Al_2_O_6_Cl_2_·10H_2_O)) was also formed in the cured APC residues at 50 °C and 70 °C because its formation is favored at relatively high temperatures (Um et al. 2014). Hydrocalumite is formed by a reaction between calcium aluminate hydrate and chloride (Bobirică et al. 2018). This formation immobilizes chloride ions (Cl^−^) and releases hydroxide ions (OH^−^) (Eq. (6)), suggesting the impact of Pb solubility. Immobilization of Cl^−^ might reduce the leaching potential of Pb owing to reduction of forming Pb-chloride complex (Weibel et al. 2018). Because of the release of OH^−^, Pb is precipitated as Pb(OH)_2_ at pH 8–13 and Pb(OH)_2_ is dissolved at pH 13–14 (Van Herck et al. 2000; Zhang et al. 2008). In these curing experiments, the pH of the leachate was 11–12. It is considered that hydrocalumite formation has positive effects on Pb immobilization when considering the impact of Cl^−^ and OH^−^ ions. Ettringite and hydrocalumite formed in MSWI residues can immobilize Pb by substitution and absorption reactions (Piantone et al. 2004; Yu et al. 2020). Part of the Pb in the cured APC residues may be immobilized by these secondary minerals. Further, calcite, which is formed by natural carbonation during the drying process, possibly reduced Pb leachability in cured APC residues via absorption on their surface and/or incorporation into their crystal structure (Wang et al. 2010). As mentioned in the “Impact of curing of air pollution control residues on lead immobilization” section, the leaching amount of Pb was reduced by 18–67% even without diatomite addition. Secondary minerals formed by hydration and carbonation reactions contribute to Pb immobilization to a certain extent.

6 $${3\mathrm{CaO Al}}_{2}{\mathrm{O}}_{3}{\mathrm{ Ca}(\mathrm{OH})}_{2} {12\mathrm{H}}_{2}{\mathrm{O }+ 2\mathrm{Cl}}^{-}\to {3\mathrm{CaO Al}}_{2}{\mathrm{O}}_{3} {\mathrm{CaCl}}_{2} {12\mathrm{H}}_{2}{\mathrm{O }+ 2\mathrm{OH}}^{-}$$

**Fig. 9:**
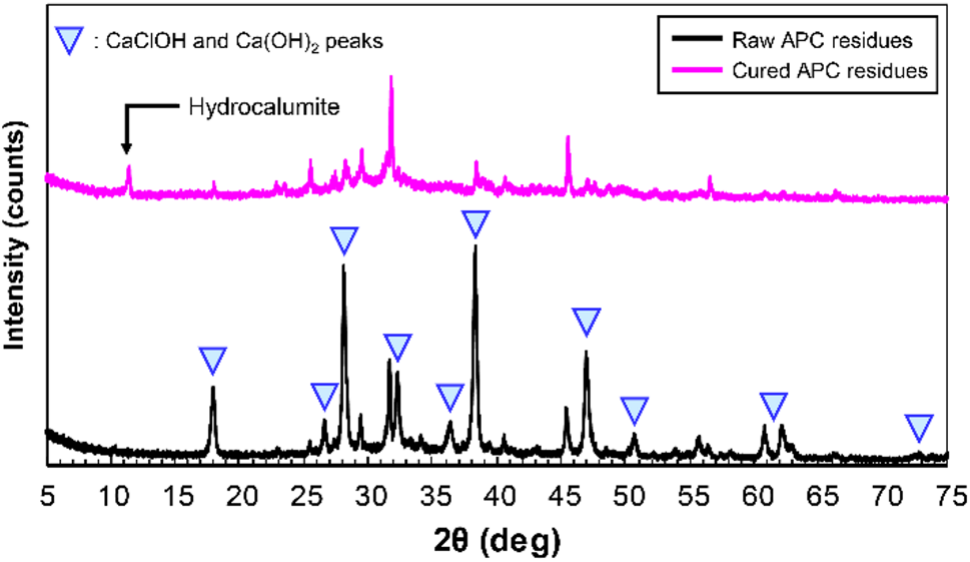
XRD patterns of raw and cured APC residues with 10% diatomite at 70 °C for 14 days

**Fig. 10:**
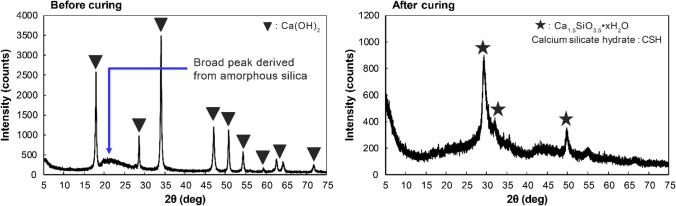
XRD patterns of mixture of Ca(OH)_2_ and diatomite before and after curing at 70 °C for 14 days

Figure 9 shows the XRD patterns of 14-day cured APC residues following the addition of 10% diatomite at 70 °C compared with the raw APC residue sample. The peak intensities of CaClOH and Ca(OH)_2_ significantly decreased after curing with diatomite, and were not decreased after curing without diatomite (see Fig. 8). This indicates that CaClOH and Ca(OH)_2_ reacted with diatomite for the pozzolanic reaction. In this case, the pH of leachate only decreased from 11.7 to 11.2 (see Fig. 5) because CaClOH and Ca(OH)_2_ remained in cured APC residues even after the pozzolanic reaction. It appeared that the pozzolanic reaction could not be completed by the curing condition, and the added diatomite was not sufficient to consume all of the CaClOH and Ca(OH)_2_ in APC residues. In addition, new peaks of pozzolanic phases in the cured APC residues were not confirmed, as they were below the detectable level of XRD analysis or incomplete crystallization (Assi et al. 2020). Therefore, a mixture of Ca(OH)_2_ reagent and diatomite (1:1) following the addition of distilled water was cured at 70 °C for 14 days to confirm the formation of pozzolanic phases, and then the cured mixture was analyzed by XRD. The results showed the formation of C-S–H (Ca_1.5_SiO_3.5_·xH_2_O) (Fig. 10). The XRD results also showed that hydrocalumite was still formed as a secondary mineral regardless of diatomite addition (see Fig. 9). Hydrocalumite is formed by the hydration reaction between Al, Ca, and Cl derived from APC residues via the curing process (Liu et al. 2018). However, hydrocalumite formation might be limited by the low content of Al in the raw APC residues (Table 3). Because of the limited formation, decreasing peak intensities of CaClOH and Ca(OH)_2_ were not clearly observed in the cured APC residues without the addition of diatomite. According to these results, secondary minerals such as hydrocalumite and pozzolanic phases, such as C-S–H gel, are formed together in APC residues cured with diatomite. Because the peak intensities of CaClOH and Ca(OH)_2_ significantly decreased after APC residues were cured with diatomite, the amount of C-S–H gel formation appeared to exceed the amount of hydrocalumite formation. Even though C-S–H gel formation was not detectable in the XRD analysis, the amount was sufficient to immobilize almost all the Pb in APC residues.

The state of Pb in C-S–H gel seems to be important for the stability of immobilization. C-S–H gel is a mixture of crystalline and amorphous phases with a layered structure (Guo et al. 2017). Vespa et al. (2014) suggested that C-S–H gel has three potential positions for metal immobilization in its structure: (1) surface complexation, (2) uptake in the interlayer, and (3) incorporation in the Ca or Si sheets. The stability of Pb immobilization in the interlayer and sheets might be higher than that on the surface. However, XRF and XRD analyses used in this study were insufficient to investigate the Pb state in C-S–H gel. For example, XRF analysis does not provide the chemical speciation of the measured elements and XRD analysis only provides a signal from the crystalline fraction of the sample. Therefore, X-ray absorption fine structure (XAFS) spectroscopy using synchrotron radiation is a promising analytical technique for further investigation because it can determine the local coordination chemistry, electric state, and local atomic structure around the absorbing atoms in complex compounds (Contessi et al. 2021).

### Chemical forms of lead estimated by sequential chemical extraction

**Fig. 11:**
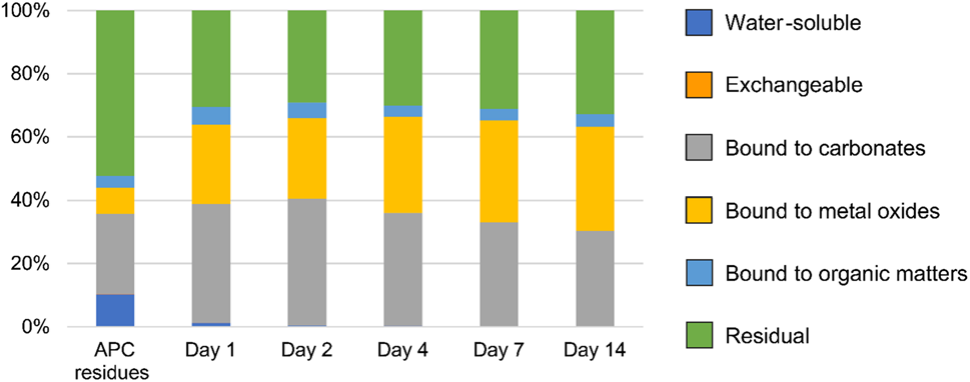
Fractionation of Pb in raw and cured APC residues following diatomite addition (10% diatomite cured at 70 °C for 14 days)

Figure 11 shows the results of sequential chemical extraction. The chemical forms of Pb in APC residues changed over the curing period via the addition of 10% diatomite at 70 °C. The water-soluble Pb in raw APC residues, which was easily leached out in leaching tests, was 10.3%. Residual Pb, which is insoluble in aqua regia (HNO_3_:HCl = 1:3) and finally remains in the stable mineral fraction, was the main fraction in raw APC residues (52.4%). After 1 day of curing, the water-soluble and residual Pb contents decreased to 1.2% and 30.5%, respectively. In contrast, carbonate- and metal oxide-bound fractions increased as the curing time increased. The carbonate-bound fraction might have increased due to carbonation via atmospheric CO_2_. Residual Pb might be decreased by release from the matrix through the curing process because the aluminosilicate matrix of APC residues dissolves under alkaline conditions (Dermatas et al. 2005). Eventually, the leaching amount of Pb was reduced owing to the decrease in water-soluble Pb via the pozzolanic reaction.

The main Pb fractions in the cured APC residues were bound to carbonate, metal oxide, and residual fractions. Because the residual fraction of Pb is incorporated into the aluminosilicate matrix in APC residues (Huang et al. 2007), it is less soluble even at strong acid conditions. Cerussite (PbCO_3_) and Pb oxides are probably classified as carbonate-bound and metal oxide–bound fractions (Funatsuki et al. 2012). Bayuseno and Schmahl (2011) suggested that Pb in MSWI fly ash is less soluble in water because almost Pb exists as crystalline phases such as Pb oxides. Even with the low solubility, Pb leaching might be controlled by Pb oxides above pH 9 (Astrup et al. 2006). Lead leaching is also controlled by cerussite at pH 8–10 (Eighmy et al. 1995). In the presence of high Cl content derived from CaClOH, KCl, and NaCl in APC residues, Pb might be dissolved by PbCl_2_ formation at pH 0–5 as well as Pb_2_(OH)_3_Cl formation above pH 12 (Zhang et al. 2008; Du et al. 2018). Thus, Pb leaching depends on the release of controlling minerals under different pH conditions (Du et al. 2018). However, the controlling minerals were different in these previous studies because metal speciation in APC residues depends on the chemical potential of elements and flue gas compositions such as contents of chlorine, moisture, sulfur, and inorganic particulates (Chiang et al. 1997; Chen et al. 1998; Youcai et al. 2004; Jiao et al. 2013). In this study, Pb solubility at pH 11–12 was investigated to confirm the effect of diatomite addition on Pb immobilization (see Fig. 7). Thus, Pb solubility over a wide pH range should be further investigated to understand the Pb state in C-S–H gel as well as the stability of Pb immobilization.

## Conclusion

Diatomite, which is mainly comprised of amorphous silica, was used as a pozzolanic material for Pb immobilization in APC residues instead of cement. The curing experiment showed that the leaching amount of Pb from the cured APC residues was reduced as the curing time and temperature increased. Even without diatomite addition, the leaching amount of Pb after 14 days of curing was reduced by 18–67% owing to secondary mineral formation (ettringite, hydrocalumite, and calcite). When diatomite was added to APC residues, it reacted with Ca(OH)_2_/CaClOH to form pozzolanic phases such as C-S–H gel. As water-soluble Pb in APC residues was reduced by the pozzolanic reaction, the leaching amount of Pb was reduced by 99% after 14 days of curing with 10% diatomite at 70 °C. Consequently, the leaching amount of Pb dropped below 0.3 mg/L (Japanese criteria for landfill disposal). Thus, the mineralogical characteristics (crystalline and amorphous phases) of APC residues played a substantial role in Pb immobilization.

Although 10% diatomite was added to the APC residues in this study, the volume is supposed to be lower than that in cement-based S/S (Sun et al. 2016). From the viewpoint of landfill management, this would also reduce the use of chemical agents while suppressing volume. Even though further studies are required, this method holds potential for the treatment of APC residues as it is low-cost and environmental-friendly.

## Data Availability

All data generated or analyzed during this study are included in this published article.
